# A Comparison of Different Implant Site Preparation Techniques in Low-Density Bone: An Ex-Vivo Study

**DOI:** 10.7759/cureus.70318

**Published:** 2024-09-27

**Authors:** Abdulkarim Sheikh ibrahim, Mohamad Hassan Jaafo

**Affiliations:** 1 Department of Oral and Maxillofacial Surgery, Faculty of Dentistry, Damascus University, Damascus, SYR

**Keywords:** densah burs, expanders, implant stability quotient, insertion torque, low-density bone, magnetic mallet

## Abstract

Introduction: Osseointegration is considered a prerequisite for the long-term success of dental implants, and many researchers have considered the stability of the implant when placed in the bone. Many techniques include undersized drilling, densifying burs, magnetic mallets, and expanders. These methods have led to higher initial insertion torque values. The present study aims to evaluate the effectiveness of expanders, densifying burs, and magnetic mallet methods for preparing the implant site in low-density bone and compare them in terms of achieving good initial implant stability.

Materials and methods: The present study was conducted in an ex vivo animal model using bovine rib bones. This study was performed on 20 bovine ribs; each rib had four implant site preparations divided into four groups according to the drilling method: a control group of the conventional technique (n=20), the expanders group (n=20), the densification burs group (n=20), and the magnetic mallet group (n=20). The measured values were Primary Insertion Torque and Implant Stability Quotient (ISQ).

Results: The highest average insertion torque was in the magnetic Mallet group, where the average was 43.75 N/cm^2^, followed by the burs group, where the average was 43.00 N/cm^2^, then the expanders group with an average of 32.80 N/cm^2^, then the conventional preparation group with 19.30 N/cm^2^ as the lowest average among the study groups. The highest ISQ mean was in the burs group, where the mean was 80.30, followed by the magnetic Mallet group, where the mean was 80.20, then the expanders group with a mean of 68.90, then the conventional preparation group with 50.10 as the lowest mean among the study groups.

Conclusion: Within the limitation of this study, we conclude that all methods used were better than conventional preparation in both ISQ and insertion torque, with the magnetic mallet group outperforming the insertion torque and the Densah burs outperforming the ISQ.

## Introduction

Osseointegration is considered a prerequisite for the long-term success of dental implants, and many researchers have considered the stability of the implant when placed in the bone, known as primary stability, to be a critical factor in achieving successful osseointegration [1].

This primary stability depends on the bone’s quality, the shape of the retainer, and the drilling protocol used to prepare the implant site [2].

When the bone quality is poor at the dental implant site, such as in the posterior region of the maxilla, achieving adequate osseointegration and long-term success may be very difficult [3,4].

Many systems and procedures have been proposed to assess bone quality and predict outcomes, as the mechanical behavior of the bone is an important factor in achieving osseointegration [5].

The classification proposed by Zarb and Lekholm for bone density assessment has been popular in the medical literature. Although this method provides valuable information about bone density, it has recently been considered subjective. Schwarzetel introduced a more objective concept of using computed tomography (CT) scanning in the preoperative quantitative assessment of patients requiring dental implant treatment [6].

Dental implant placement in low-density bone (type IV) has a higher chance of failure than implants placed in other bone types. This type of bone is often found in the posterior region of the maxilla and is usually associated with a higher implant failure rate [7].

The surface and geometry of the dental implant are among the most studied variables, considering their impact on implant stability [8].

However, it is not clear how the implant site preparation protocol affects the implant success rate. Several surgical protocols have been proposed to enhance the survival rate of implants placed in low-density bone [9].

The conventional stepwise drilling technique is the classic method for preparing the implant site, using gradually increasing diameter twisted drills rotating at speeds of 800 to 1500 rounds per minute (rpm) under sufficient irrigation to avoid raising the bone temperature [10].

Many techniques include undersized drilling, densifying burs, magnetic mallets, and expanders. These methods have led to higher initial insertion torque values, which are indicators of improved initial implant stability and may increase the chances of implant success rate [11].

The present study aims to evaluate the effectiveness of expanders, densifying burs, and magnetic mallet methods for preparing the implant site in low-density bone and compare them in terms of achieving good initial implant stability.

## Materials and methods

The present study was conducted in an ex vivo animal model using bovine rib bones. The animals were not used exclusively for this study, and the bones were collected as they were disposable as waste material, so ethical approval was not necessary. Bovine ribs were collected on the day of the slaughter. This study was performed on 20 bovine ribs; each rib had four implant site preparations divided into four groups according to the drilling method: a control group of the conventional technique (n=20), the expanders group (n=20), the densification burs group (n=20), and the magnetic mallet group (n=20). No samples were lost during sample processing and analysis.

Bovine ribs were prepared by the removal of soft tissues, and then cone beam computed tomography (CBCT (Vatech, Italy) was performed to determine their radiographic density. Ribs with a density of type IV (less than 350 Hounsfield units) according to the Lekholm & Zarb [12] classification were selected with a height greater than or equal to 12 mm and a minimum width of 6 mm.

The sample was calculated based on the data of a pilot study and used G*power software, where α was (0.05), power was (0.95), and the effect size was (2.31). The results were 5 samples in each group, and we raised to 20 to increase the power of the study.

Implant sites preparation

Four implant sites were prepared in each bovine rib after trimming 1 mm from its top to remove the cortical bone. They were selected according to the sample inclusion criteria (bone density and dimensions). Each implant site was prepared according to the method followed in each group.

**Figure 1:**
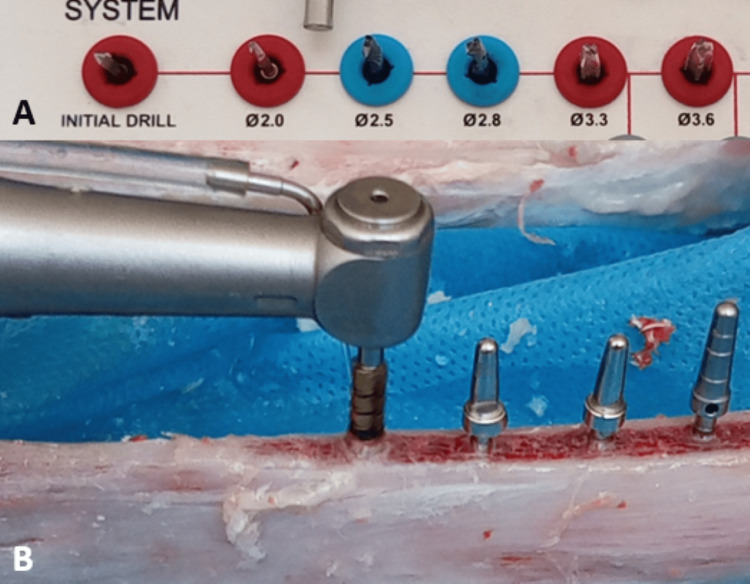
The conventional technique (A: The used kit, B: Drilling using the conventional technique)

The control group was the conventional surgical technique group, which was prepared according to the recommendations of the dental implant manufacturer (AnyOne, MEGAGEN, Korea) (Figure 1). The preparation starts with the initial drill, which is the pilot drill with a diameter of 2 mm and reaches a length of 10 mm, then the second drill with a diameter of 2.5 mm, then the third drill with a diameter of 2.8 mm, then the fourth drill with a diameter of 3.3 mm, and finally the drill with a diameter of 3.6 mm. The implant was inserted into the implant site with a diameter of 4 mm and a length of 10 mm.

**Figure 2:**
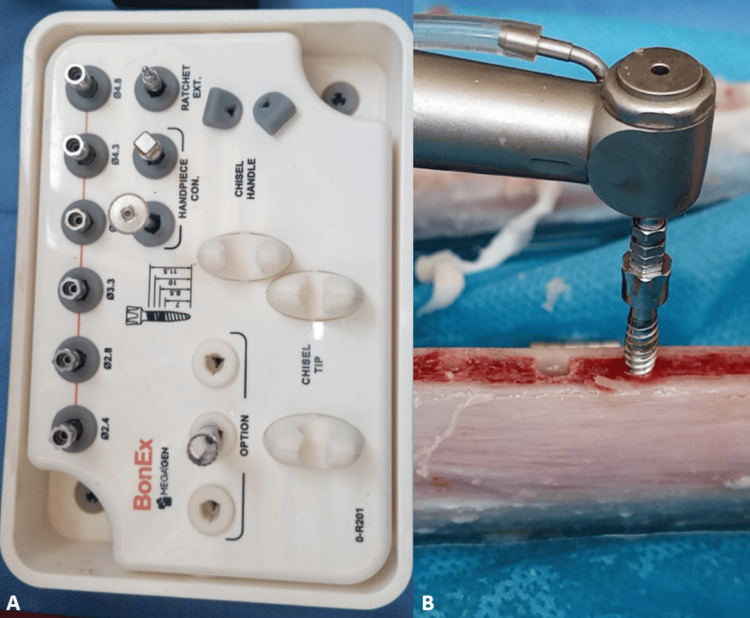
The expanders method (A: The BonEx kit, B: Drilling using the expanders method)

The first study group was the bone expansion technique using bone expanders (BonEx, MEGAGEN, Korea) (Figure 2), which was performed in the following order: starting with the initial drill (Pilot Drill) with a diameter of 2 mm, then moving to the first expander with a diameter of 2.4 mm, then the second expander with a diameter of 2.8 mm, and finally the expander with a diameter of 3.3 mm. The implant was inserted into the implant site with a diameter of 4 mm and a length of 10 mm.

**Figure 3:**
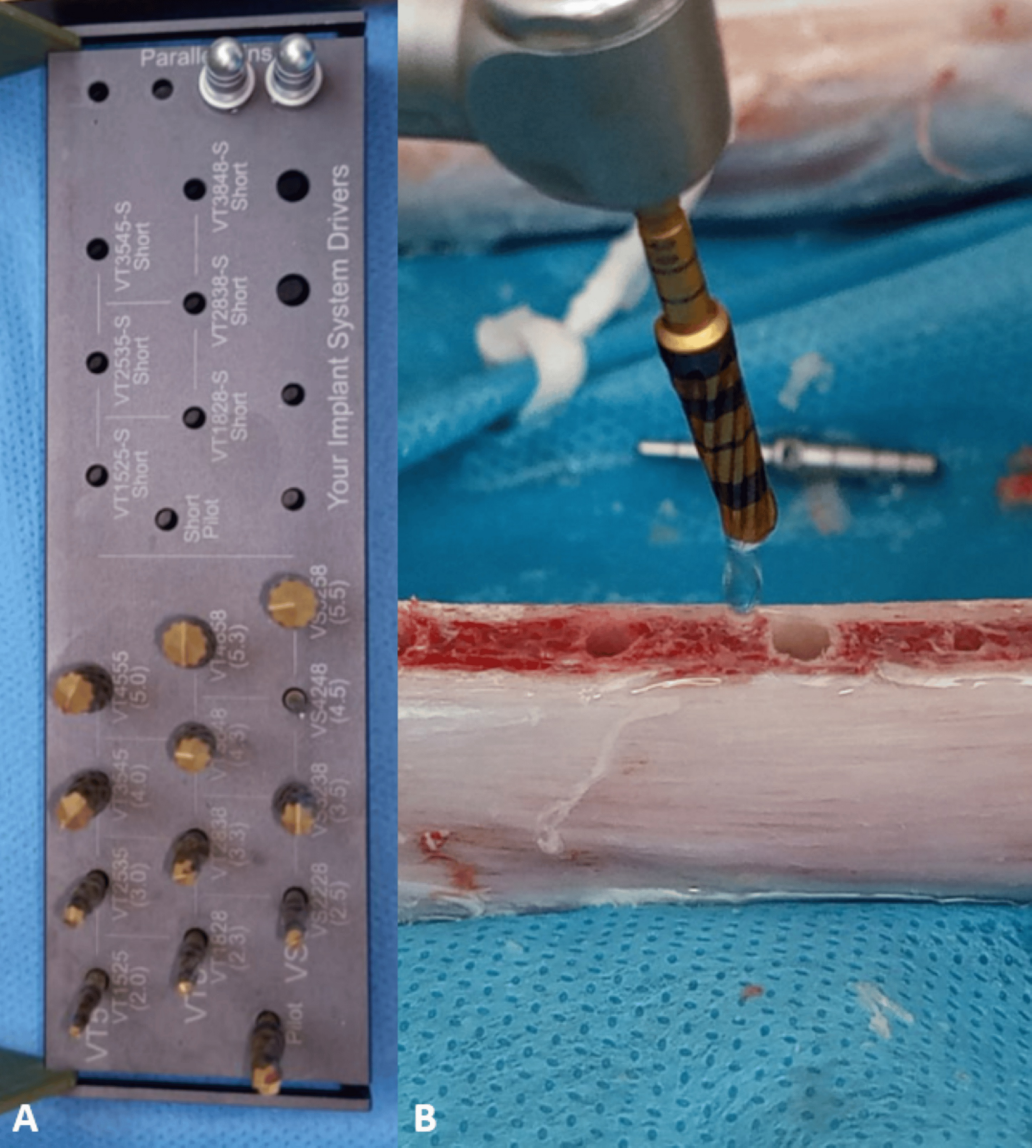
The Densah burs technique (A: The Densah kit, B: Drilling using Densah burs)

The second study group was the bone densification using Densah densification burs (Densah, USA) (Figure 3), where this technique starts with the initial drill (Pilot Drill) with a diameter of 2 mm, then the second drill with a diameter of 2.3 mm, then the third drill with a diameter of 3 mm, and finally the drill with a diameter of 3.3 mm. The implant was inserted into the implant site with a diameter of 4 mm and a length of 10 mm.

**Figure 4:**
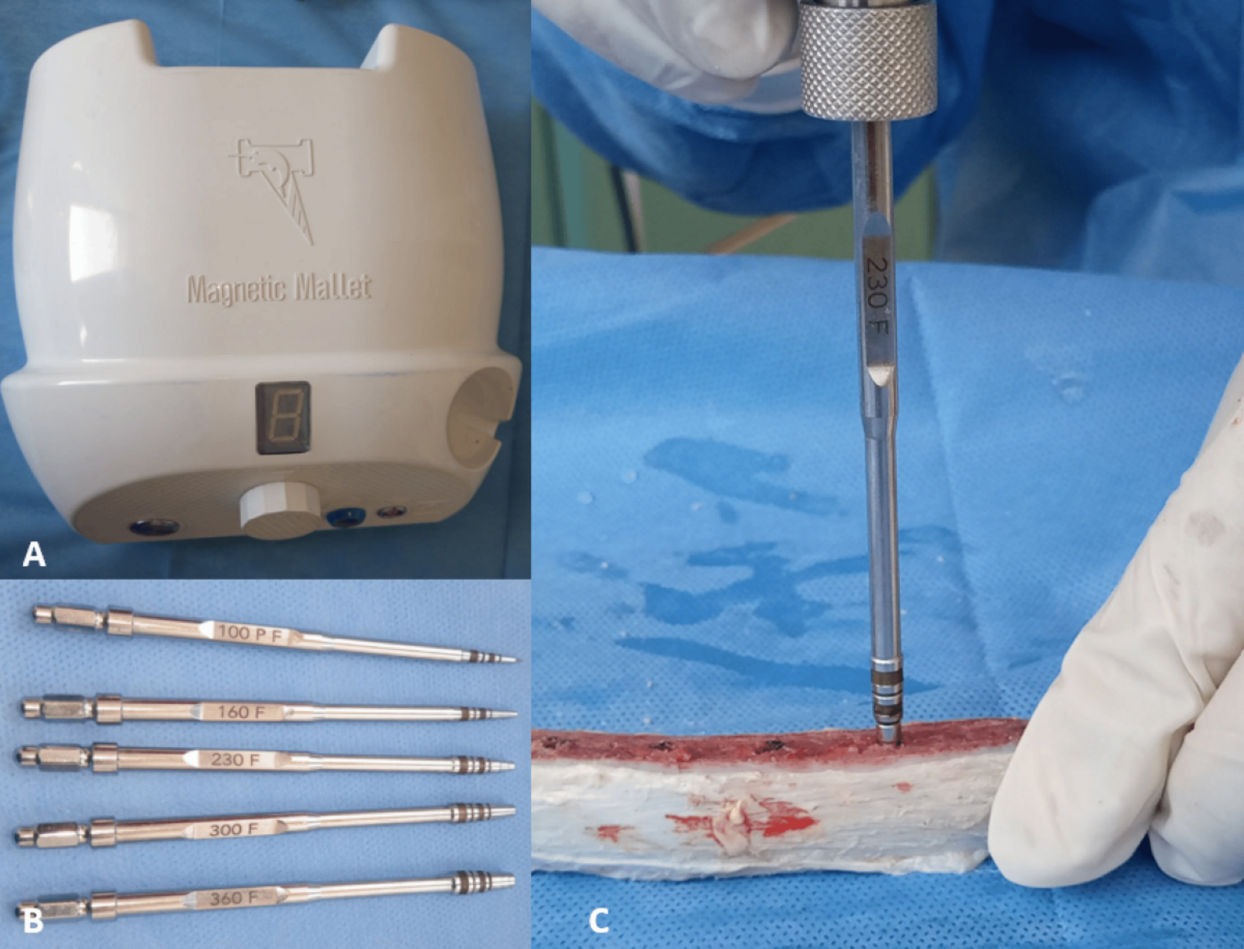
The magnetic mallet technique (A: The magnetic mallet device, B: The magnetic mallet drills, C: Drilling using the magnetic mallet)

The third study group was the Magnetic Mallet (Meta Ergonomica Srl; Turbigo MI, Italy) (Figure 4), where the implant site was prepared using a mallet with a head diameter of 1 mm, then a 1.6 mm head diameter, then a 2.3 mm head diameter, and finally a 3 mm head diameter. The implant was inserted into the implant site with a diameter of 4 mm and a length of 10 mm.

Primary insertion torque

The insertion torque values required to place the implant at the bone level were recorded in Newtons per square centimeter (N/cm^2^) by recording the value measured on a surgical motor (Craft, Korea).

Implant stability quotient (ISQ)

**Figure 5:**
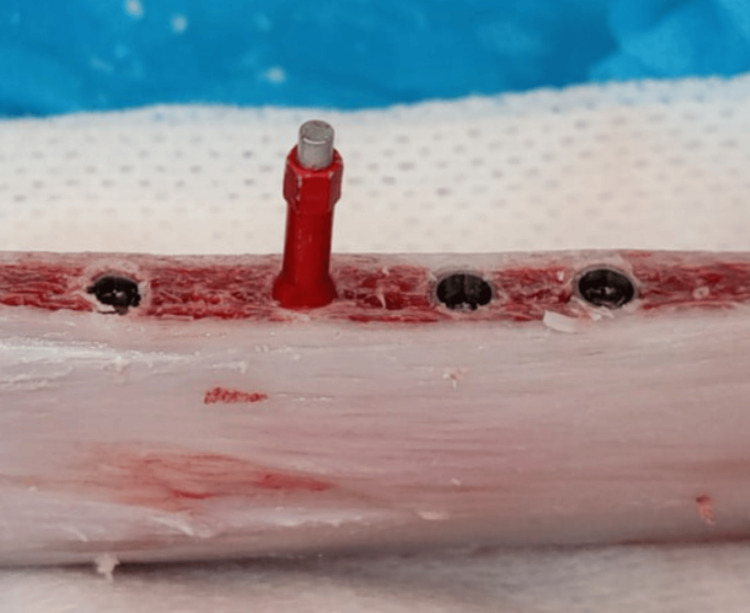
Measuring the implant stability quotient

The MEGA ISQ device (MEGA ISQ, MEGAGEN, Korea) was used to measure the implant stability quotient (ISQ) by inserting the smart peg into the dental implant (Figure 5) immediately after the implant was inserted, and then the head of the device was placed in four random locations around the smart peg to calculate the average of the recorded ISQ values. 

Statistical analysis

The data was analyzed using SPSS v.25 (SPSS, IBM, USA). The one-sample Kolmogorov-Smirnov test was used to determine the distribution of data, a one-way ANOVA test was used to study the differences between the group means, and post-hoc comparisons with Turkey's corrections were made between each of the two groups to find the differences accurately.

## Results

The study sample included 20 bovine ribs. Four implant sites were prepared in each rib, and each implant site was prepared differently according to the four study groups.

Primary insertion torque

Table 1 shows that the highest average insertion torque was in the magnetic Mallet group, where the average was 43.75 N/cm^2^, followed by the burs group, where the average was 43.00 N/cm^2^, then the expanders group with an average of 32.80 N/cm^2^, then the conventional preparation group with 19.30 N/cm^2^ as the lowest average among the study groups.

**Table 1:** Descriptive data for primary insertion torque values (N/cm2) for each study group

Group	Count	Mean (N/cm^2^)	Standard deviation
Conventional	20	19.30	4.497
Expanders	20	32.80	2.745
Burs	20	43.00	3.811
Magnetic Mallet	20	43.75	3.754

The one-sample Kolmogorov-Smirnov test was used to determine the distribution of data, where all groups took the normal distribution as indicated by the results of the Kolmogorov-Smirnov test, and the p-value in all groups was higher than 0.05.

A one-way ANOVA test was used to study the differences between the group means, which shows the existence of significant differences between the group means at a significance level of less than 0.05 (Table 2).

**Table 2:** One-way ANOVA test results to study the differences in the primary insertion torque between the group means

Squares Sum	Freedom Degree	Squares Mean	ANOVA Value	p-Value
8204.54	5	1640.91	128.39	<0.001

Post-hoc comparisons with Turkey's correction were made between each of the two groups to find the differences accurately (Table 3).

**Table 3:** Post-hoc comparisons with Turkey's correction between each two groups

Group I	Group J	Mean of Difference (I-J)	Standard Error	p-Value
Conventional	Expanders	-13.500	1.131	<0.001
	Burs	-23.700	1.131	<0.001
	Magnetic Mallet	-24.450	1.131	<0.001
Expanders	Burs	-10.200	1.131	<0.001
	Magnetic Mallet	-10.950	1.131	<0.001
Burs	Magnetic Mallet	-0.750	1.131	0.986

A significant difference was found in the primary insertion torque between the conventional preparation groups and the expanders group in favor of the expanders group, between the conventional preparation groups and the expanders group in favor of the expanders group, and between the conventional preparation groups and the magnetic Mallet in favor of the magnetic Mallet group. Thus, we found that all groups outperformed the conventional preparation group with a significant difference (<0.001).

A significant difference was found in the primary insertion torque between the expanders groups and the burs group in favor of the expanders group and between the expanders groups and the magnetic Mallet in favor of the expanders group.

No significant differences were found between the burs group and the magnetic Mallet group.

Implant stability quotient (ISQ)

Table 4 shows that the highest ISQ mean was in the burs group, where the mean was 80.30, followed by the magnetic Mallet group, where the mean was 80.20, then the expanders group with a mean of 68.90, then the conventional preparation group with 50.10 as the lowest mean among the study groups.

**Table 4:** Descriptive data for implant stability quotient (ISQ) values for each study group

Group	Count	Mean	Standard deviation
Conventional	20	50.10	5.19
Expanders	20	68.90	4.14
Burs	20	80.30	2.94
Magnetic Mallet	20	80.20	3.66

The one-sample Kolmogorov-Smirnov test was used to determine the distribution of data, where all groups took the normal distribution as indicated by the results of the Kolmogorov-Smirnov test, and the p-value in all groups was higher than 0.05.

A one-way ANOVA test was used to study the differences between the group means, which shows the existence of significant differences between the group means at a significance level of less than 0.05 (Table 5).

**Table 5:** One-way ANOVA test results to study the differences in Implant Stability Quotient (ISQ) between the group means

Squares Sum	Freedom Degree	Squares Mean	ANOVA Value	P-Value
8204.54	5	2615.50	140.235	<0.001

Post-hoc comparisons with Turkey's correction were made between each of the two groups to find the differences accurately (Table 6).

**Table 6:** Post-hoc comparisons with Turkey's correction between each two groups

Group I	Group J	Mean of Difference (I-J)	Standard Error	P-Value
Conventional	Expanders	-18.80	1.366	<0.001
	Burs	-30.20	1.366	<0.001
	Magnetic Mallet	-30.10	1.366	<0.001
Expanders	Burs	-11.40	1.366	<0.001
	Magnetic Mallet	-11.30	1.366	<0.001
Burs	Magnetic Mallet	0.10	1.366	1.00

A significant difference was found in the ISQ between the conventional preparation groups and the expanders group in favor of the expanders group, between the conventional preparation groups and the burs group in favor of the burs group, and between the conventional preparation groups and the magnetic Mallet group in favor of the magnetic Mallet group. Thus, we found that all groups outperformed the conventional preparation group with a significant difference (<0.001).

A significant difference was found in the ISQ between the expanders group and the burs group in favor of the burs group, and between the expanders group and the magnetic Mallet group in favor of the magnetic Mallet group.

No significant differences were found in the ISQ between the burs group and the magnetic Mallet group.

## Discussion

It is clear that the need for special preparation techniques in low-density bone is beginning to take on greater importance due to the difficulty of ensuring good initial stability for implants, such as in the posterior region of the maxilla, where studies have shown that dental implants have the highest failure rate compared to other types of bone [13].

Some clinical and laboratory studies [14-17] have evaluated the effect of these different tools on bone density and implants. However, there are no studies that compare these techniques with each other in terms of their effect on implant stability.

The superiority of the magnetic mallet in primary insertion torque can be explained by the minimum bone removal compared to other methods, as the preparation required before starting the densification was 1.2 mm [18]. Densah burs were in second place, and this is also due to the lateral densification that these burs perform at the site of preparation, as both their design and their counterclockwise rotation direction ensure good densification of the bone.

Expanders ranked third, which may be explained by the fact that these expanders do not densify the bone laterally like Densah burs.

The superiority of the preparation technique using densification burs in ISQ over other methods can be explained by its densification mechanism, due to the unique design of the drills and the counterclockwise rotation, which helps in densifying the bone apically and laterally [15], which enhances the local density and increases the contact surface area between the implant and the surrounding bone, thus positively improving the level of implant stability.

The low levels of ISQ in the conventional preparation method are explained by the bone removal caused by conventional drills and thus their inability to increase the local bone density around the implants, and due to the low bone density of the recipient’s bone, the implant will not achieve high stability.

The good implant stability values in the preparation group using the magnetic mallet that was close to those achieved by the densification burs can also be explained by the lateral and apical bone density achieved by the magnetic mallet for preparing the implant site and minimal bone removal and thus increased contact surface area between the implant and the bone.

The good implant stability values ​​in the preparation group using bone expanders can be explained by minimal bone removal in the implant site and the lateral bone densification it produces.

Cáceres's study [17] compared conventional preparation with Densah burs in increasing the implant stability, where he prepared the implant sites in pig’s bone after slaughter. The results of the study concluded that Densah burs densification resulted in a greater insertion torque than the conventional preparation and a greater ISQ than the conventional preparation.

Jarikian's study [16] compared Densah burs and expanders in bone expansion in narrow bone ridges and concluded that both methods resulted in bone expansion and increased density without cracks or fenestrations.

Mahmoud's study [15] aimed to compare Densah burs with expanders in increasing the stability of implants in the upper premolar region. The study concluded that Densah burs were superior to expanders in insertion torque and ISQ, which is consistent with the results of the current study.

Sanad's study [14] compared the magnetic mallet with the conventional preparation in increasing the stability of implants by measuring the insertion torque and the ISQ. The study concluded that the magnetic mallet outperformed the conventional preparation in achieving greater ISQ and insertion torque.

This study has several limitations, such as being an ex-vivo study and not a clinical study, in addition to not performing a histological study of bone formation as it is an ex-vivo study.

## Conclusions

The initial stability of the implants plays an essential role in the osseointegration process and is affected negatively and positively by the bone density (the ratio of cortical and medullary bone). It is known to the researchers that the increase in cortical bone and indicators of initial stability thus play a vital role in the implant’s stability. Within the limitations of this study, we conclude that all methods used were better than conventional preparation in both ISQ and insertion torque, with the magnetic mallet group being superior in the insertion torque and the Densah burs in the ISQ.
